# Plasma, urine, and stool metabolites in response to dietary rice bran and navy bean supplementation in adults at high-risk for colorectal cancer

**DOI:** 10.3389/fgstr.2023.1087056

**Published:** 2023-02-15

**Authors:** Emily B. Hill, Bridget A. Baxter, Brigitte Pfluger, Caroline K. Slaughter, Melanie Beale, Hillary V. Smith, Sophia S. Stromberg, Madison Tipton, Hend Ibrahim, Sangeeta Rao, Heather Leach, Elizabeth P. Ryan

**Affiliations:** ^1^ Department of Pediatrics, Section of Nutrition, School of Medicine, University of Colorado, Aurora, CO, United States; ^2^ Department of Environmental and Radiological Health Sciences, College of Veterinary Medicine and Biomedical Sciences, Colorado State University, Fort Collins, CO, United States; ^3^ Rollins School of Public Health, Emory University, Atlanta, GA, United States; ^4^ Department of Health and Exercise Science, College of Health & Human Sciences, Colorado State University, Fort Collins, CO, United States; ^5^ Colorado School of Public Health, Colorado State University, Fort Collins, CO, United States; ^6^ Department of Medical Biochemistry, Faculty of Medicine, Zagazig University, Zagazig, Egypt; ^7^ Department of Clinical Sciences, College of Veterinary Medicine and Biomedical Sciences, Colorado State University, Fort Collins, CO, United States

**Keywords:** rice bran, navy bean, colorectal cancer, nutritional metabolomics, dietary supplementation, amino acids, lipids, bile acids

## Abstract

**Introduction:**

Dietary intake of whole grains and legumes and adequate physical activity (PA) have been associated with reduced colorectal cancer (CRC) risk. A single-blinded, two-arm, randomized, placebo-controlled pilot trial was implemented to evaluate the impact of a 12-week dietary intervention of rice bran + navy bean supplementation and PA education on metabolite profiles and the gut microbiome among individuals at high risk of CRC.

**Methods:**

Adults (n=20) were randomized 1:1 to dietary intervention or control. All participants received PA education at baseline. Sixteen study foods were prepared with either heat-stabilized rice bran + navy bean powder or Fibersol^®^-2 as a placebo. Intervention participants consumed 30 g rice bran + 30 g navy bean powder daily; those in the control group consumed 10 g placebo daily. Non-targeted metabolite profiling was performed by UPLC-MS/MS to evaluate plasma, urine, and stool at 0, 6, and 12 weeks. Stool was also analyzed for primary and secondary bile acids (BAs) and short chain fatty acids (SCFAs) by UPLC-MS/MS and microbial community structure *via* 16S amplicon sequencing. Two-way ANOVA was used to compare differences between groups for metabolites, and mixed models were used to compare differences between groups for BAs, SCFAs, and alpha and beta diversity measures of microbial community structure.

**Results:**

Across biological matrices, the intervention resulted in changes to several amino acid and lipid metabolites, compared to control. There was a 2.33-fold difference in plasma (p<0.001) and a 3.33-fold difference in urine (p=0.008) for the amino acid S-methylcysteine at 12 weeks. Fold-differences to 4-methoxyphenol sulfate in plasma and urine after 6 and 12 weeks (p<0.001) was a novel result from this combined rice bran and navy bean intervention in people. A 2.98-fold difference in plasma (p=0.002) and a 17.74-fold difference in stool (p=0.026) was observed for the lipid octadecenedioylcarnitine at 12 weeks. For stool BAs, 3-oxocholic acid was increased at 12 weeks compared to control within a subset of individuals (mean difference 16.2 ug/uL, p=0.022). No significant differences were observed between groups for stool SCFAs or microbial community structure.

**Discussion:**

Dietary intake of rice bran + navy beans demonstrates beneficial modulation of host and gut microbial metabolism and represents a practical and affordable means of increasing adherence to national guidelines for CRC control and prevention in a high-risk population.

## 1 Introduction

Colorectal cancer (CRC) is the third most common cancer diagnosis and the third leading cause of death from cancer in the United States, with estimates of over 1.4 million men and women in the United States living with a CRC diagnosis in 2022 (1, 2). CRC rates continue to increase among individuals younger than 50 years old, attributable in part to lifestyle factors such as poor quality dietary patterns and lack of physical activity, though 95% of CRC diagnoses are among those greater than 50 years old (1–3). While preclinical and clinical studies have demonstrated greater consumption of whole grains, plant fibers from fruits, vegetables, and legumes, and several micronutrients to be associated with a decreased risk of CRC, greater dietary intakes of red and processed meats and alcohol, and low intakes of micronutrients have been associated with an increased risk of CRC (3–8). Greater adherence to physical activity recommendations has also been shown in several studies to reduce risk of CRC (9). Thus, targeted interventions may help to improve dietary intake and physical activity patterns for CRC prevention, particularly among high-risk populations (10, 11).

Rice bran (a whole grain component) and navy beans (a legume) are functional foods containing an abundance of bioactive compounds that may confer a chemoprotective effect when consumed in adequate amounts (12–16). Research demonstrates as much as a 40% reduction in precancerous adenomatous polyps with consumption of brown rice 1-2 times per week, while dry bean intake of 1.5 cups per week has likewise been associated with a reduced risk of polyp development (17–20). Similarly, physical activity interventions focused on increases in moderate to vigorous physical activity have been associated with reduced risk of colorectal cancer and development of polyps in the general population as well as among cancer survivors (21, 22). Together, these data suggest a combined functional food dietary intervention incorporating both rice bran and navy beans together with PA education may lead to a synergistic effect that can reduce future risk of CRC.

It is hypothesized that rice bran and navy beans may influence disease risk through impacts on both the host metabolome and modulation of the gut microbiota. We have demonstrated that the rice bran and navy bean metabolomes contain amino acids, lipids, and phytochemicals that may alter the plasma, urine, and stool metabolomes after consumption, potentially leading to direct or indirect effects on future CRC risk (23–25). These effects may be explained through several mechanisms underlying risk of CRC development, including changes in gut microbiota composition and function. These alterations may correct dysbiosis, which if uncorrected can lead to diminished concentrations of beneficial short chain fatty acids (SCFAs) and increased production of pro-inflammatory and pro-carcinogenic secondary bile acids (BAs) (26–31). Indeed, the high fiber content of these foods fed separately in studies supports fermentation in the large intestine that can increase bacterial production of SCFAs such as acetate, propionate, and butyrate (32, 33). Additionally, increased dietary fiber intake alters the BA profile by hindering BA reabsorption and cholesterol uptake, suggesting a potential mechanism by which changes in dietary intake may synergistically act to improve colon health (34–36). Increased dietary fiber intake has also demonstrated the ability to increase abundance of beneficial microbes and lower levels of toxic microbial metabolites such as N-nitroso compounds and phenolics such as p-cresol (37, 38).

Notably, our team has demonstrated rice bran *or* navy bean supplementation can modulate the human plasma, urine, and stool metabolomes as well as the gut microbiome in CRC survivors (39–42). Thus, the aims of this 12-week study were to: (1) assess the impact of a *combined* rice bran + navy bean placebo-controlled dietary intervention on the plasma, urine, and stool metabolome for adults at risk for CRC; (2) identify changes in specific amino acid and lipid metabolites associated with CRC risk; and (3) identify changes in the stool microbiome and functional products (SCFAs, BAs) of the gut microbiota. It was hypothesized that the combined rice bran + navy bean dietary intervention would produce beneficial shifts in plasma, urine, and stool metabolites, with variable excretion of SCFAs and primary and secondary BAs as related to changes in metrics of microbial diversity and relative abundances of dietary responsive taxa in the human gut microbiota.

## 2 Materials and methods

### 2.1 Study design and dietary interventions

**Figure 1:**
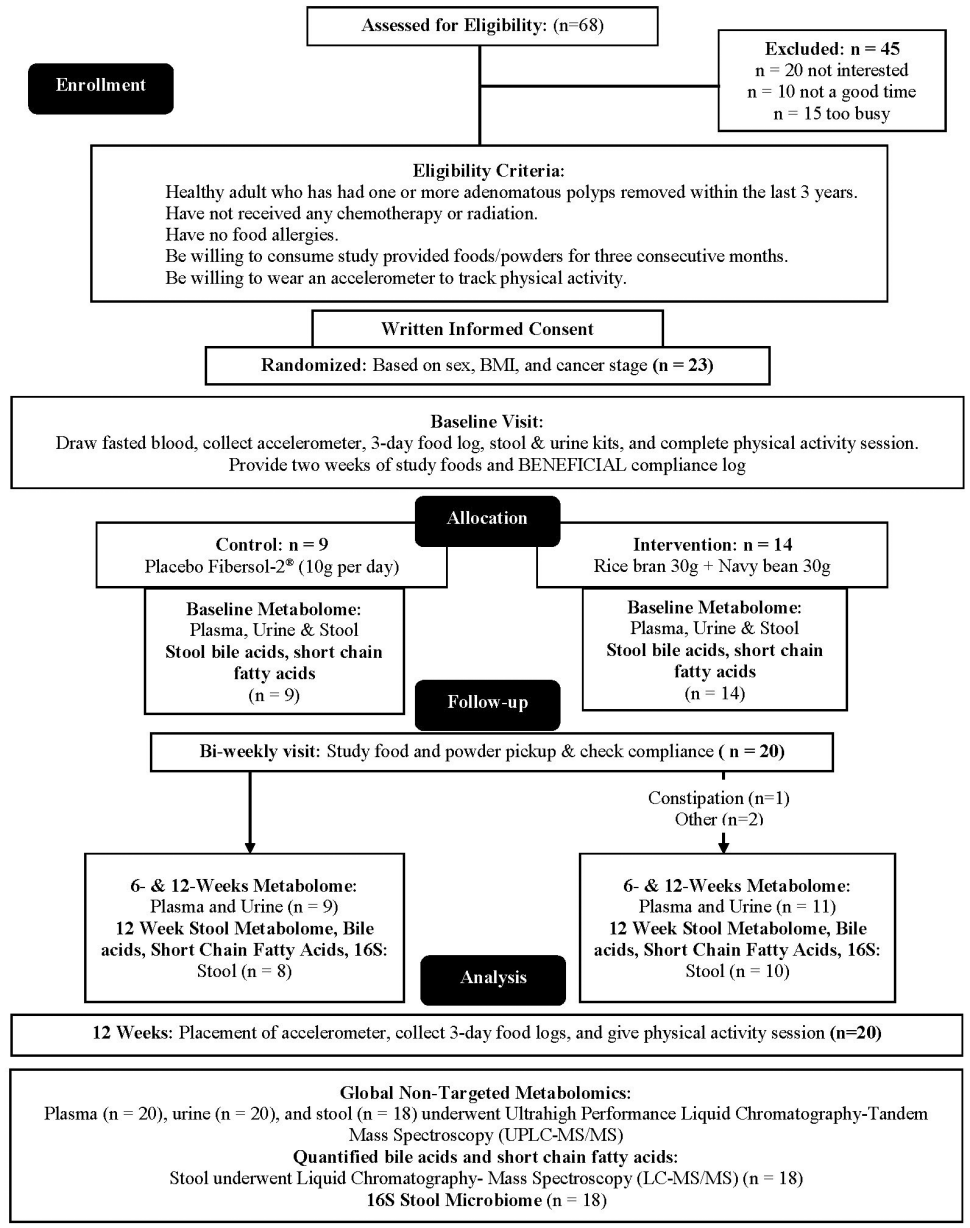
CONSORT flow diagram: A single-blinded, two-arm, randomized, placebo-controlled pilot trial was implemented to evaluate impacts of dietary intake of rice bran + navy beans on the plasma, urine, and stool metabolome and gut microbiome. Adults at risk for colorectal cancer (CRC) were randomized 1:1 to either a 12-week rice bran + navy bean intervention or placebo control. The plasma, urine, and stool metabolomes and gut microbiome were assessed at baseline (week 0), mid-intervention (week 6), and post-intervention (week 12) for those who completed the intervention and provided biospecimens. Within and between group changes were assessed at 6 and 12 weeks (n = 20).

Adults at risk for CRC were recruited for this single-blinded, two-arm, randomized, placebo-controlled pilot trial. Individuals with at least one adenomatous polyp removed at routine colonoscopy were targeted due to the evidence suggesting increased risk of incident CRC may be mitigated by changes in lifestyle factors such as diet (43–46). Full methods were previously described (47). Briefly, Beans/Bran Enriching Nutritional Eating For Intestinal Health & Cancer Including Activity for Longevity (BENEFICIAL) eligibility criteria included: (1) healthy adults (≥18 years of age) who had one or more adenomatous polyps removed within the previous 3 years; (2) had not received adjuvant treatment such as chemotherapy or radiotherapy with their surgical removal of polys; (3) had no history of food allergies; (4) were willing to consume study provided foods/powders for 12 weeks; (5) were not lactating or pregnant. All participants were blinded to the study arms. Recruitment took place through the UCHealth-North Cancer Center (Fort Collins, CO, USA) and through the Colorado State University email listserv community. **Figure 1** shows the CONSORT diagram. Prior to dietary intervention, stratified block randomization was completed to assign participants 1:1 by sex and cancer stage to one of the two study groups. The body mass index (BMI) were then classified as normal (18.5-24.9 kg/m^2^), overweight (25.0-29.9 kg/m^2^), or obese (≥30 kg/m^2^). BMI and underlying pre-existing health conditions (e.g., diabetes, cardiovascular, Crohn’s disease) were not matched between the two study groups. All participants reported having at least 3 polyps removed at routine colonoscopy, but neither total number of polyps nor location were confirmed by pathology reports. **Table 1** illustrates the participant characteristics at baseline. BMI for the intervention arm was significantly different from the control group at baseline. In this 12-week intervention, participants assigned to the rice bran + navy bean group consumed 30 g heat-stabilized rice bran + 30 g of cooked navy beans in powder form [daily intake of two study foods and one study powder that each contained 10 g rice bran + 10 g navy bean powder (47)]. The control group received placebo study foods (without addition of rice bran or navy beans) and one study powder of 10 g Fibersol^®^-2, as previously described (47). Participants were free-living for the remainder of their daily caloric intake needs. 

**Table 1:** Participant baseline demographics.

Characteristics (n = 20)	Control (n = 9)	Intervention (n = 11)	P-Value
**Age (years),** (mean ± SD)	58.9 ± 7.8	59.2 ± 9.3	0.94
**Sex**			0.67
Males (%)	4 (44%)	6 (55%)	
Females (%)	5 (56%)	5 (45%)	
**BMI (kg/m^2^),** (mean ± SD)	29.5 ± 2.1	25.9 ± 3.5	0.02
Normal weight (20-24.9 kg/m^2^)	0 (0%)	5 (45%)	
Overweight (25-29.9 kg/m^2^)	5 (56%)	4 (37%)	
Obese (≥30 kg/m^2^)	4 (44%)	2 (18%)	
**Cancer stage**			0.83
Stage 0 <6 mm	7 (78%)	9 (82%)	
Stage 1 >6 mm	2 (22%)	2 (18%)	

Data presented in mean ± standard deviation, percent, or number; mm indicates millimeters.

### 2.2 Blood, urine, and stool sample collection

Three scheduled study visits were completed at week 0 (baseline), week 6 (mid-intervention), and week 12 (end of intervention). Fasted blood samples (n=20) were collected into 4 mL ethylene-diamine-tetra-acetic acid (EDTA) collection tubes and kept immediately on ice until centrifugation at 1,500 rpm for 10 min to extract plasma. Plasma was aliquoted into study ID-labeled tubes and stored at -80°C until processed for metabolomics analysis. First morning void urine was self-collected by participants (n=20) in a study ID-labeled container at baseline, week 6, and week 12. Urine samples were aliquoted and stored at -80°C until processed for metabolomics analysis. Stool samples were self-collected by participants (n=18) into study ID-labeled containers within 24 hours of study visits at baseline and week 12 only. The stool sample was divided by study staff into two aliquots. One aliquot containing raw stool was immediately transferred to a study ID-labeled tube for SCFA analysis and 16S amplicon sequencing prior to freezing, and the remaining frozen stool was lyophilized for fecal metabolomics and BA analysis. Both aliquots (frozen and lyophilized) were stored at -80°C until analysis.

### 2.3 Plasma, urine, and stool non-targeted metabolomics analysis

Metabolon, Inc. (Morrisville, NC, USA) performed the non-targeted metabolomics on plasma, urine, and stool samples. Briefly, 80% methanol was added to plasma, urine, and lyophilized stool samples. The samples were shaken for 2 min, centrifuged at 12,000 rpm for 10 min at 4°C, and then dried under nitrogen prior to analysis. Quality control samples were also prepared, including a pooled sample to serve as a technical replicate and an extracted water sample to serve as a process blank. Internal standards were spiked into each analyzed sample. Plasma, urine, and stool metabolite extracts were analyzed using ultra-high performance liquid chromatography-tandem mass spectrometry (UHPLC-MS/MS) with positive and negative ion mode electrospray ionization (ESI), as described below (48).

Non-targeted analysis of metabolites was completed for plasma, urine, and stool using a Waters ACQUITY ultra-performance liquid chromatography (UPLC) and a Thermo Scientific Q-Exactive high resolution/accurate mass spectrometer (MS) interfaced with a heated electrospray ionization (HESI-II) source and Orbitrap mass analyzer operated at 35,000 mass resolution. The sample extracts for each biological matrix were reconstituted in solvents compatible to each of the four methods described below. Each reconstitution solvent contained a series of standards at fixed concentrations to ensure injection and chromatographic consistency. One aliquot was analyzed using acidic positive ion conditions and a method chromatographically optimized for hydrophilic compounds. In this method, the extract was gradient eluted from a C18 column (Waters UPLC BEH C18-2.1x100 mm, 1.7 µm) using water and methanol (MeOH), containing 0.05% perfluoropentanoic acid (PFPA) and 0.1% formic acid (FA). A second aliquot was analyzed using acidic positive ion conditions and a method chromatographically optimized for hydrophobic compounds. In this method, the extract was gradient eluted from a C18 column using water, MeOH, acetonitrile (ACN), 0.05% PFPA, and 0.01% FA and was operated at an overall higher organic content. A third aliquot was analyzed using basic negative ion optimized conditions using a separate dedicated C18 column. The basic extracts were gradient eluted from the column using MeOH and water with 6.5 mM ammonium bicarbonate at pH 8. The fourth aliquot was analyzed *via* negative ionization following elution from a HILIC column (Waters UPLC BEH Amide 2.1x150 mm, 1.7 µm) using a gradient consisting of water and ACN with 10 mM ammonium formate at pH 10.8. The MS analysis alternated between MS and data-dependent MS^n^ scans using dynamic exclusion. The scan range varied slightly between methods but covered 70-1000 *m/z*.

Raw data were extracted, peak-identified, and QC processed by Metabolon utilizing Microsoft’s.net technologies. Peaks were quantified using area under the curve. Compounds were identified by comparison to library entries of purified standards or recurrent unknown entities. Metabolon maintains a library based on authentic standards with retention time/index (RI), mass to charge ratio (*m/z)*, and chromatographic data (including MS/MS spectral data) on all molecules present in the library. Identifications were based upon three criteria: (1) RI within a narrow RI window; (2) accurate mass match to the library ± 10 ppm; and (3) MS/MS forward and reverse scores between experimental data and authentic standards.

### 2.4 Targeted quantification of stool BAs and SCFAs

Stool metabolite extraction and targeted BA and SCFA quantification was performed at the Colorado State University Analytical Resources Core: Bioanalysis and Omics facility. Ultra-high-performance liquid chromatography tandem mass spectrometry was performed for five primary BAs (cholic, taurocholic, glycocholic, chenodeoxycholic, and glycochenodeoxycholic acids) and 12 secondary BAs (deoxycholic, ursodeoxycholic, lithocholic, nutriacholic, 7alpha-Hydroxy-3-oxo-5beta cholanoic, hyodeoxycholic, 3-oxocholic, 3alpha,6beta,7beta-Trihydroxy-5b-cholanoic, glycodeoxycholic, taurodeoxycholic, 3beta-hydroxy-5-cholenoic, and sulfolithocholic acids), following published methods and described below (49). Gas chromatography mass spectrometry (GC-MS) was performed for assessment of six SCFAs (butyric, propionic, isobutyric, isovaleric, valeric, and acetic acids), following published methods described below and are listed in **Supplementary Table 1** (49).

#### 2.4.1 BA analysis

Lyophilized stool (10 mg) samples were arranged in randomized order and processed in 0.1 mL of 0.1 M NaOH (pH 13) for 1 hour at 60°C; samples were vortexed every 20 min. 200 µL of freezer-cold (-20°C) 100% ACN (spiked with an internal standard mix at 400 ng/mL) was added to each sample prior to vortexing at 4°C for 30 min. Samples were left at -80°C overnight. Precipitate was collected after centrifugation at 17,000 x g for 30 min at 4°C the following morning. Supernatant (180 μL) was transferred to a 200 μL glass vial insert and vials were stored at -80°C until UPLC-MS/MS analysis. Authentic standards of all target analytes were prepared from dry stock at a concentration of 1 mg/mL in 100% ACN. A master mix of all target analytes was prepared in 100% ACN at a concentration of 0.1 mg/mL. Internal standards (Taurocholic acid-d5 and Deoxycholic acid-d4) were prepared at the same concentrations. Dilution series were made in 30% ACN, 70% 100 mM NaOH spiked with internal standards mix at 400 ng/mL. Starting concentration was 5,000 ng/mL. Low point concentration was 0.25 ng/mL, with an internal standard-only zero point to calculate standard deviation of the background signal.

Targeted analysis was performed on a Waters Acquity UPLC coupled to a Waters Xevo TQ-S triple quadrupole mass spectrometer. Chromatographic separations were carried out on a Waters T3 stationary phase (1 x 50 mm, 1.7 μM) column. Mobile phases were ACN (B) and water with 0.1% FA (A). The analytical gradient was as follows: time = 0 min, 30% B; time = 0.65 min, 30% B; time = 2.85 min, 97% B; time = 3.5 min, 97% B; time 3.55 min, 30% B; time = 5 min, 30% B. Flow rate was 800 μL/min and injection volume was 0.5 μL. Samples were held at 4°C in the autosampler, and the column was operated at 45°C. Study-specific pooled quality control samples were prepared and injected between every four analytical samples throughout the experiment. The mass spectrometer was operated in selected reaction monitoring (SRM) mode optimized for each analyte by direct injection of individual standards. Ions were monitored in negative ionization mode with the capillary voltage set to 1.8 kV. Source temperature was 150°C and desolvation temperature 550°C. Desolvation gas flow was 1000 L/hr, cone gas flow was 150 L/hr, and collision gas flow was 0.2 mL/min. Nebulizer pressure (nitrogen) was set to 7 bar. Argon was used as the collision gas. A calibration curve was generated using authentic standards for each compound and their corresponding stable isotope labeled internal standards in neat solution.

Raw data files were imported into Skyline open source software for data extraction (50). Peak areas for target compounds were normalized to the peak areas of the internal standards for each sample. Normalized peak areas were exported for absolute quantitation *via* linear regression using the calibration curve for each compound. Limits of detection (LOD) and limits of quantitation (LOQ) were calculated as three times or 10 times the standard deviation of the blank divided by the slope of the calibration curve, respectively (51, 52).

#### 2.4.2 SCFA analysis

Frozen stool (20 mg) samples were arranged in randomized order for extraction, and 340 μL of cold 3 M HCl and 60 μL of internal standard solution containing 1 mg/mL of ^13^C_2_-acetic acid (Sigma-Aldrich, St. Louis, MO) and 0.5 mg/mL of ^13^C_4_-sodium butyrate (Santa Cruz Biotechnology, Dallas, TX) were added. Samples were vigorously shaken for 30 min, followed by sonication for 10 min in a cold water bath, and then centrifuged at 15,000 x g for 15 min at 4°C. Supernatants (200 μL) were recovered and added to 350 μL methyl tert-butyl ether (MTBE), followed by vortexing for 5 sec twice. Approximately 60 μL of the top MTBE layer were recovered after centrifugation at 3,000 x g for 5 min at 4°C and stored at 4°C until analysis.

The MTBE extracts of SCFAs (1 μL) were injected into a Thermo Trace 1310 gas chromatograph (Thermo, Waltham, MA) coupled to a Thermo ISQ-LT mass spectrometer, at a 5:1 split ratio. Samples were arranged in randomized order for injection along with seven quality controls that were generated from a pooled sample extract and injected after every six samples. The inlet was held at 240°C. The SCFA separation was achieved on a 30 m DB-WAXUI column (0.25 mm ID, 0.25 μm film thickness; J&W, Folsom, CA). Oven temperature was held at 100°C for 0.5 min, increased by 10°C/min to 175°C, then increased by 40°C/min to 240°C, and held at 240°C for 3 min, with a total run time of ~12.6 minutes. Helium carrier gas flow was held at 1.2 mL/min. Temperatures of transfer line and ion source were both held at 250°C. Selected ion monitoring (SIM) mode was used at a rate of 10 scans/sec under electron impact mode.

Data were processed using Chromeleon software (version 7.2.8; Thermo, Folsom, CA). The internal standard ^13^C_2_-acetic acid was used to quantify acetic and propionic acids; ^13^C_4_-sodium butyrate was used to quantify other SCFAs. The coefficient of variation (CV) of quality controls was 1.0% to 3.6%. Linearity with R^2^ > 0.997 was obtained from all calibration curves. LOD and LOQ were calculated using the standard deviation of blanks and the slope of calibration curve.

### 2.5 Stool 16S amplicon sequencing

Frozen stool samples were thawed on ice and homogenized prior to DNA extraction with the MoBio PowerSoil Kit (MoBio Laboratories Inc.) per manufacturer protocols. The V4 hypervariable region of the 16S rRNA gene was amplified and sequenced on the Illumina MiSeq platform according to the Earth Microbiome Project standards using the 515F and 806R (53–55). A total of 3,464,689 raw single-end FASTQ formatted forward sequence reads represented by 36 samples were imported into the Quantitative Insights Into Microbial Ecology 2 (QIIME 2) (56). Alignment to the SILVA database of microbial genomes was used for taxonomic assignment (45).

### 2.6 Statistical analysis

Plasma, urine, and stool non-targeted metabolites were normalized using median-scaled relative abundance, whereby metabolites were quantified by the relative abundance and median-scaled to 1. Repeated-measures ANOVA was used for within group comparisons from baseline to 6 and 12 weeks. Between groups, a Welch two-sample *t* test and two-way ANOVA were performed. Significantly different metabolites within groups (fold change) or between groups (fold difference), were calculated by dividing the median-scaled abundance of each metabolite in the rice bran + navy bean group at 6 and/or 12 weeks by their baseline at week 0 or that of the Fibersol^®^-2 group, respectively. Statistically different metabolites between the rice bran + navy bean and Fibersol^®^ – 2 groups at baseline were removed from the between group analyses. False discovery rate (*q*-value) was calculated to account for multiple comparisons. No comparisons across biological matrices were completed. Standard statistical analyses were performed in ArrayStudio on log transformed data with statistical significance of adjusted p-values ≤0.05.

Taxonomically assigned sequencing reads from 16S amplicon sequencing were analyzed for relative abundance using the Phyloseq R package (46). Alpha diversity was compared using observed and Shannon index metrics (47). Beta diversity was compared using unweighted Unifrac distances for principal coordinate analysis ordination (48).

A mixed model was used for analysis of quantified stool BAs, SCFAs, and alpha and beta diversity to compare within group changes from baseline to 12 weeks and between group differences at 12 weeks. The analysis was adjusted for repeated measures. All data are represented in ug/uL (parts per billion (ppb)) for each metabolite. Statistical significance was defined at alpha ≤0.05. SAS v9.4 (SAS Institute Inc., Cary, NC) was used for all statistical analyses.

## 3 Results

### 3.1 Dietary modulation of plasma, urine, and stool metabolome

There were 1002 plasma (n=20), 1001 urine (n=20), and 962 stool (n=18) metabolites identified in non-targeted analyses across all time points. From this metabolome, we focused on changes to amino acids and lipids. We measured 213 amino acid and 459 lipid metabolites in plasma, 275 amino acid and 186 lipid metabolites in urine, and 219 amino acid and 369 lipid metabolites in stool across all time points. Forty-nine metabolites (34 amino acids and 15 lipids) were removed from analysis for changes with the intervention due to being significantly different between study arms at baseline. Six amino acids were removed from plasma, 11 from urine, and 17 from stool prior to assessing changes with dietary intervention over time. Four lipids were removed from plasma, 2 from urine and 9 from stool. **Supplementary Table 2** shows area counts for plasma, urine, and stool amino acid and lipid metabolites classified into metabolic sub-pathways for control and intervention participants across all time points.

### 3.2 Plasma, urine, and stool amino acids modulated by rice bran + navy bean intervention

**Supplementary Table 3** lists 94 amino acid metabolites (33 unique to plasma, 60 in urine, 27 in stool, and 26 in more than one biological matrix) that were significantly modulated within either intervention or control groups at week 6 and/or week 12 compared to baseline. All data are presented as mean fold-change in the median-scaled relative abundance of each metabolite. Within the intervention group, 15 plasma (12 increased and 3 decreased) and 12 urine (11 increased and 1 decreased) amino acid metabolites were significantly different at week 6 weeks when compared to baseline. At 12 weeks, 18 plasma (15 increased, 3 decreased), 28 urine (all increased), and 14 stool (2 increased and 12 decreased) metabolites were significantly different. Of the amino acid metabolites that changed within the intervention group, 11 plasma (10 increased and 1 decreased) and 5 urine (all increased) amino acid metabolites were significantly different at both weeks 6 and 12.

Eighteen amino acid metabolites in plasma, urine, and/or stool demonstrated significant differences between the intervention and control groups at week 6 and/or week 12 (**Table 2**). Data are presented as the mean fold-difference between the intervention and the control group.

**Table 2:** Plasma, urine, and stool amino acid metabolite changes after 6 and 12 weeks in rice bran + navy bean intervention compared to the placebo-control group.

Amino Acid Metabolites	Metabolite Fold-Differences Between Intervention (Rice bran + Navy beans) and Control (Fibersol^®^-2)									
	Plasma				Urine				Stool	
	Week 6	p-value	Week 12	p-value	Week 6	p-value	Week 12	p-value	Week 12	p-value
Histidine Metabolism										
1-methyl-4-imidazoleacetate	0.87	0.601	1.11	0.994	0.81	0.588	1.20	0.789	**0.32**	0.038
Lysine Metabolism										
Pipecolate	**2.00**	0.006	**4.15**	<0.001	1.65	0.262	**2.27**	0.016	0.80	0.457
Phenylalanine Metabolism										
N-succinyl-phenylalanine	–	–	–	–	**1.36**	0.034	0.98	0.661	0.56	0.734
Phenylpyruvate	1.21	0.126	**1.46**	0.007	1.23	0.503	1.14	0.597	**0.30**	0.041
2-hydroxyphenylacetate	1.44	0.348	1.19	0.472	1.17	0.106	**1.46**	0.012	0.78	0.616
Tyrosine Metabolism										
phenol sulfate	**2.40**	0.012	1.11	0.447	**2.50**	0.006	**1.46**	0.012	2.46	0.535
phenol glucuronide	–	–	–	–	**3.05**	0.026	0.78	0.276	–	–
4-methoxyphenol sulfate	**14.48**	<0.001	**3.07**	0.001	**12.55**	<0.001	**3.52**	<0.001	–	–
N-formylphenylalanine	0.95	0.776	0.97	0.948	**0.23**	0.009	1.90	0.200	0.98	0.670
Tryptophan Metabolism										
Anthranilate	–	–	–	–	**2.48**	<0.001	0.95	0.623	–	–
Indoleacetate	**1.64**	0.030	1.27	0.425	1.15	0.776	1.23	0.356	1.13	0.399
Leucine, Isoleucine, and Valine Metabolism										
N-methylleucine	–	–	–	–	**5.64**	0.007	**24.29**	<0.001	**3.62**	0.025
2,3-dihydroxy-2-methylbutyrate	1.45	0.063	**1.76**	0.003	1.42	0.061	**1.86**	0.024	–	–
Methionine, Cysteine, SAM, and Taurine Metabolism										
S-methylcysteine	**1.82**	0.012	**2.33**	<0.001	1.39	0.542	**3.33**	0.008	–	–
S-methylcysteine sulfoxide	**2.17**	0.029	**2.69**	0.006	1.02	0.803	1.92	0.163	–	–
Urea cycle; Arginine and Proline Metabolism										
N2,N5-diacetylornithine	**2.06**	0.001	**2.22**	0.001	**1.89**	0.023	**2.01**	0.009	1.60	0.347
N,N,N-trimethyl-alanylproline betaine (TMAP)	**1.18**	0.046	1.03	0.684	0.92	0.761	0.94	0.531	32.97	0.783
Creatine Metabolism										
Creatine	**0.67**	0.018	0.85	0.312	0.41	0.164	**0.44**	0.037	9.28	0.399

The median scaled relative abundance was used to calculate means for each metabolite in plasma, urine, and stool. Each matrix metabolite abundance was analyzed separately. Statistically significant fold-differences are **bolded** (p ≤ 0.05).

**Figure 2:**
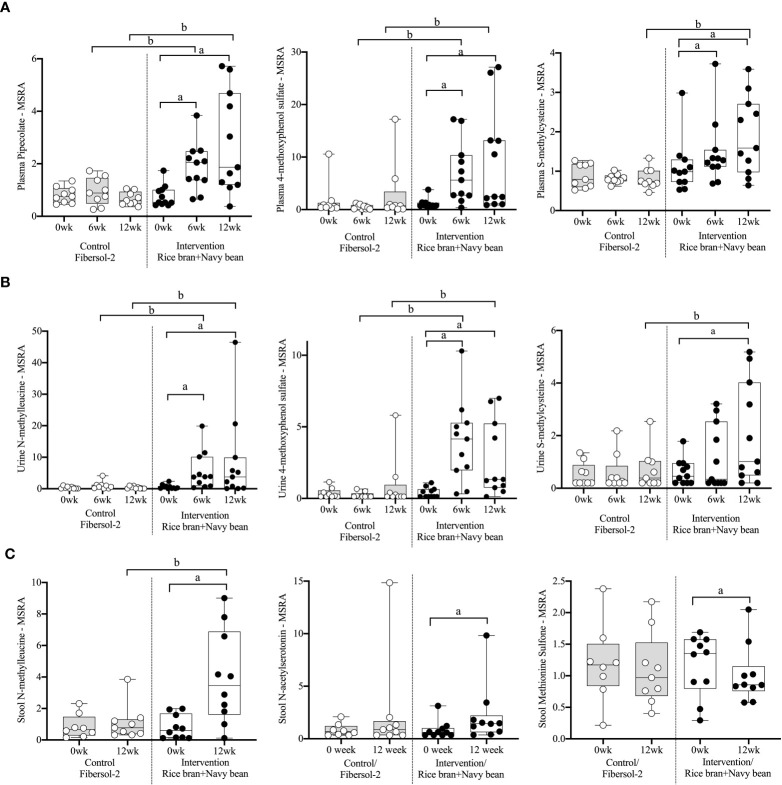
Median-scaled relative abundance (MSRA) of selected plasma, urine, and stool amino acid metabolites that changed significantly within and between groups from week 0 to week 6 and week 12 post dietary intervention. **(A)** Plasma, **(B)** Urine, **(C)** Stool a = significant fold-change, b = significant fold-difference (*p ≤ 0.05*).

Eight plasma amino acid metabolites increased and one decreased for the rice bran + navy bean group at 6 weeks compared to control. One amino acid involved in lysine metabolism, two in tyrosine metabolism, one in tryptophan metabolism, two in methionine, cysteine, S-adenosyl methionine (SAM) and taurine metabolism, and two involved in the urea cycle were increased, while one amino acid involved in creatine metabolism was decreased. These differences were maintained at 12 weeks for five amino acids, including pipecolate, 4-methoxyphenol sulfate, S-methylcysteine, S-methylcysteine sulfoxide, and N2,N5-diacetylornithine. **Figure 2A** illustrates the three plasma amino acid metabolites that demonstrated significant within and between group differences at both week 6 and week 12.

Seven urine amino acid metabolites increased for the rice bran + navy bean group at 6 weeks compared to control, including one involved in phenylalanine metabolism, three in tyrosine metabolism, one in tryptophan metabolism, one in leucine, isoleucine and valine metabolism, and one involved in the urea cycle. Conversely, one amino acid involved in tyrosine metabolism decreased at week 6 when compared to the control group. Four of these amino acids remained elevated in intervention compared to control at 12 weeks, including phenol sulfate, 4-methoxyphenol sulfate, N-methylleucine, and N2,N5-diacetylornithine. **Figure 2B** illustrates the three urine amino acid metabolites that demonstrated significant within and between group differences at both week 6 and week 12.

One stool amino acid metabolite, N-methylleucine, increased for the rice bran + navy bean group at 12 weeks compared to control. Two amino acid metabolites, 1-methyl-4-imidazoleacetate and phenylpyruvate, decreased in intervention compared to control at this time point. **Figure 2C** illustrates that one metabolite, N-methylleucine, increased in abundance within the intervention group at 12 weeks when compared to baseline and was significantly different from the control group at 12 weeks.

### 3.3 Plasma, urine, and stool lipids modulated by rice bran + navy bean intervention

**Supplementary Table 4** lists 197 lipids (105 unique to plasma, 45 in urine, 58 in stool, and 11 in more than one biological matrix) that were significantly modulated within either intervention or control at week 6 and/or week 12 compared to baseline. All data are presented as mean fold-change in the median-scaled relative abundance of each metabolite. Within the intervention group, 29 plasma (16 increased, 13 decreased) and 26 urine (25 increased, 1 decreased) lipid metabolites were significantly different at 6 weeks when compared to baseline. At 12 weeks, 58 plasma (7 increased, 51 decreased), 14 urine (all increased), and 24 stool (12 increased, 12 decreased) lipid metabolites were significantly different. Of the lipid metabolites that changed within the intervention group, 12 plasma (4 increased, 8 decreased) and 8 urine (all increased) lipid metabolites were significantly different at both weeks 6 and 12.

Forty-four lipid metabolites in plasma, urine, and/or stool demonstrated significant differences between the intervention and control groups at week 6 and/or week 12 (**Table 3**). Data are presented as the mean fold-difference between the intervention and the control group.

**Table 3:** Plasma, urine, and stool lipid metabolite changes after 6 and 12 weeks in rice bran + navy bean intervention compared to the placebo-control group.

Lipid Metabolites	Metabolite Fold-Differences Between Intervention (Rice bran + Navy beans) vs Control (Fibersol^®^-2)									
	Plasma				Urine				Stool	
	Week 6	p-value	Week 12	p-value	Week 6	p-value	Week 12	p-value	Week 12	p-value
Fatty Acid, Dicarboxylate										
Pimelate (C7-DC)	–	–	–	–	**1.62**	0.047	0.85	0.646	**2.02**	0.027
hexadecanedioate (C16-DC)	1.34	0.152	**1.78**	0.017	–	–	–	–	**2.09**	0.022
2-hydroxysebacate	0.84	0.147	0.86	0.406	1.61	0.138	0.95	0.767	**2.29**	0.047
octadecenedioate (C18:1-DC)*	**2.63**	0.006	**3.68**	0.005	–	–	–	–	0.85	0.843
tridecenedioate (C13:1-DC)*	0.78	0.516	0.52	0.112	**0.48**	0.028	1.04	0.748	1.22	0.393
Fatty Acid, Amino										
2-aminoheptanoate	**1.63**	0.030	1.36	0.135	0.98	0.808	1.37	0.284	0.58	0.284
N-acetyl-2-aminooctanoate*	1.64	0.099	1.54	0.292	**1.72**	0.010	1.31	0.178	–	–
Fatty Acid Metabolism (Acyl Carnitine, Medium Chain)										
octanoylcarnitine (C8)	**1.32**	0.049	1.01	0.932	0.83	0.292	0.88	0.591	–	–
decanoylcarnitine (C10)	**1.48**	0.020	0.89	0.756	2.66	0.138	0.92	0.873	–	–
laurylcarnitine (C12)	**1.42**	0.021	1.00	0.802	0.92	0.798	0.97	0.993	2.71	0.398
Fatty Acid Metabolism (Acyl Carnitine, Long Chain Saturated)										
myristoleoylcarnitine (C14:1)*	1.31	0.117	0.95	0.944	0.47	0.120	1.06	0.587	**7.61**	0.011
Fatty Acid Metabolism (Acyl Carnitine, Monounsaturated)										
eicosenoylcarnitine (C20:1)*	1.27	0.125	1.16	0.321	–	–	–	–	**3.40**	0.013
Fatty Acid Metabolism (Acyl Carnitine, Dicarboxylate)										
octadecenedioylcarnitine (C18:1-DC)*	**2.82**	0.001	**2.98**	0.002	–	–	–	–	**17.74**	0.026
Fatty Acid Metabolism (Acyl Carnitine, Hydroxy)										
(S)-3-hydroxybutyrylcarnitine	0.84	0.465	0.76	0.339	**0.49**	0.040	0.67	0.128	–	–
3-hydroxydecanoylcarnitine	**1.52**	0.028	1.15	0.497	0.74	0.313	0.93	0.889	–	–
Fatty Acid, Dihydroxy										
9,10-DiHOME	**2.16**	0.002	**2.22**	0.006	–	–	–	–	1.40	0.683
Endocannabinoid										
N-linoleoyltaurine*	**1.69**	0.035	1.13	0.506	–	–	–	–	–	–
N-oleoylserine	**1.30**	0.039	1.08	0.507	–	–	–	–	–	–
Phospholipid Metabolism										
Choline	1.03	0.807	0.99	0.691	–	–	–	–	**0.45**	0.024
glycerophosphoinositol*	**-**	–	–	–	0.56	0.228	1.06	0.275	**0.29**	0.028
trimethylamine N-oxide	**0.66**	0.047	0.87	0.597	0.54	0.053	0.78	0.256		
Phosphatidylcholine (PC)										
1,2-dioleoyl-GPC (18:1/18:1)	–	–	–	–	–	–	–	–	**0.38**	0.025
1-oleoyl-2-linoleoyl-GPC (18:1/18:2)*	**-**	–	–	–	–	–	–	–	**0.45**	0.035
1,2-dilinoleoyl-GPC (18:2/18:2)	**1.31**	0.009	1.13	0.243	–	–	–	–		
Lysophospholipid										
1-palmitoyl-GPC (16:0)	1.03	0.578	1.02	0.800	–	–	–	–	**0.52**	0.043
1-oleoyl-GPC (18:1)	1.25	0.079	1.14	0.305	–	–	–	–	**0.44**	0.026
1-linoleoyl-GPC (18:2)	**1.32**	0.022	1.19	0.116	–	–	–	–	**0.42**	0.017
1-linolenoyl-GPC (18:3)*	1.34	0.140	1.20	0.474	–	–	–	–	**0.35**	0.044
1-palmitoyl-GPI (16:0)	1.38	0.342	1.16	0.409	–	–	–	–	**0.43**	0.044
1-oleoyl-GPI (18:1)	1.33	0.182	1.26	0.145	–	–	–	–	**0.42**	0.023
1-linoleoyl-GPI (18:2)*	1.29	0.103	1.31	0.082	–	–	–	–	**0.17**	0.033
Plasmalogen										
1-(1-enyl-palmitoyl)-2-linoleoyl-GPE (P-16:0/18:2)*	**1.32**	0.036	1.19	0.138	–	–	–	–		
1-(1-enyl-stearoyl)-2-oleoyl-GPE (P-18:0/18:1)	**1.30**	0.027	1.08	0.482	–	–	–	–		
1-(1-enyl-stearoyl)-2-linoleoyl-GPE (P-18:0/18:2)*	**1.26**	0.042	**1.23**	0.048	–	–	–	–		
Diacylglycerol										
palmitoleoyl-linoleoyl-glycerol (16:1/18:2) [1]*	**0.51**	0.037	0.92	0.547	–	–	–	–	1.04	0.547
Hexosylceramides (HCER)										
;glycosyl-N-(2-hydroxynervonoyl)-sphingosine (d18:1/24:1(2OH))*	**1.70**	0.014	1.31	0.200	–	–	–	–	0.99	0.686
Corticosteroids										
Cortisol	1.20	0.328	1.26	0.164	1.17	0.518	**2.13**	0.017	–	–
Cortisone	**1.24**	0.046	**1.33**	0.009	0.88	0.456	**1.69**	0.027	–	–
cortisone 21-sulfate	–	–	–	–	1.81	0.577	**4.01**	0.036	–	–
tetrahydrocortisol sulfate (1)	–	–	–	–	1.72	0.103	**2.92**	0.008	–	–
Androgenic Steroids										
11beta-hydroxyandrosterone sulfate (2)	–	–	**-**	–	**1.40**	0.037	1.53	0.094	–	–
Galactosyl Glycerolipids										
1-palmitoyl-2-linoleoyl-digalactosylglycerol (16:0/18:2)*	**-**	–	**-**	–	–	–	–	–	**0.31**	0.046
1-linoleoyl-2-linolenoyl-galactosylglycerol (18:2/18:3)*	**-**	–	**-**	–	–	–	–	–	**0.38**	0.039
1-linoleoyl-2-linolenoyl-digalactosylglycerol (18:2/18:3)*	**-**	–	**-**	–	–	–	–	–	**0.36**	0.006

The median scaled relative abundance was used to calculate means for each metabolite in plasma, urine, and stool. Each matrix metabolite abundance was analyzed separately. Statistically significant fold-differences are **bolded** (p ≤ 0.05). * Indicates compounds that have not been officially confirmed based on a standard, but are confident in its identity.

**Figure 3:**
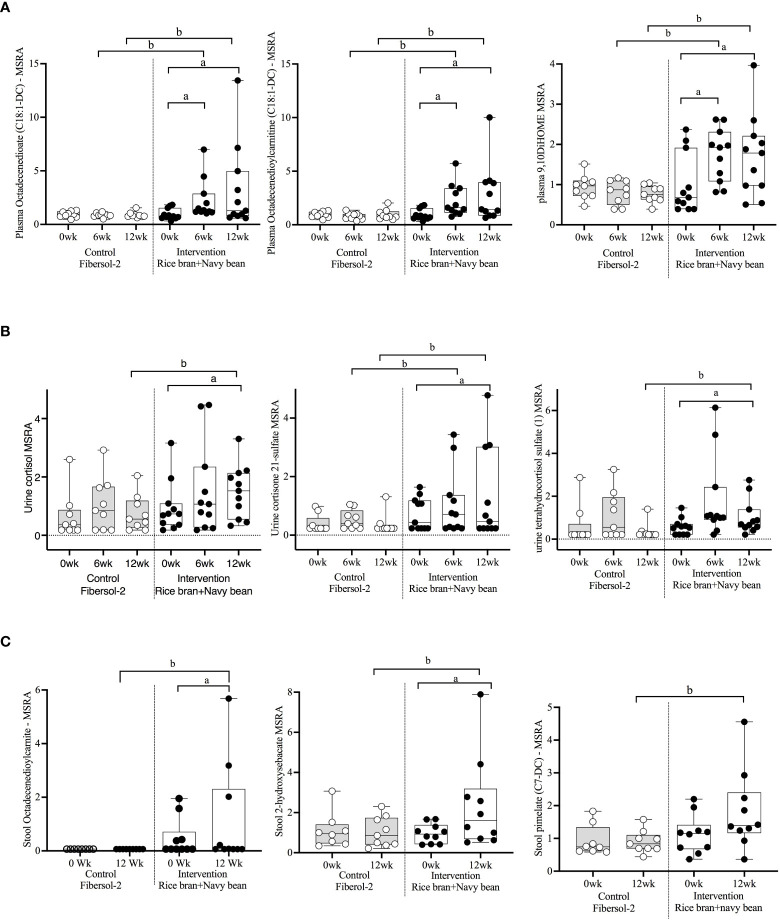
Median-scaled relative abundance (MRSA) of selected plasma, urine, and stool lipid metabolites that changed significantly within and between groups from week 0 to week 6 and week 12 post dietary intervention. **(A)** Plasma, **(B)** Urine, **(C)** Stool a = significant fold-change, b = significant fold-difference (*p ≤ 0.05*).

Seventeen plasma lipid metabolites increased at 6 weeks for the rice bran + navy bean group compared to control, including eight involved in fatty acid metabolism, two endocannabinoids, one phospatidylcholine, one lysophospholipid, three plasmalogens, one hexosylceramide, and one corticosteroid. Two plasma lipid metabolites were significantly decreased in the intervention group compared to control at 6 weeks, including one phospholipid and one diacylglycerol. Five of these changes were maintained at 12 weeks, including increased octadecenedioate (C18:1-DC), octadecenedioylcarnitine (C18:1-DC), 9,10-Dihydroxy-12-octadecenoic acid (9,10-DiHOME), 1-(1-enyl-stearoyl)-2-linoleoyl-GPE (P-18:0/18:2), and cortisone. **Figure 3A** illustrates three plasma lipid metabolites that demonstrated significant within and between group differences at both week 6 and week 12.

Three urine lipid metabolites increased at 6 weeks for the rice bran + navy bean group compared to control, including one fatty acid dicarboxylate, one amino fatty acid, and one androgenic steroid. Two urine fatty acid lipid metabolites significantly decreased compared to the control group at 6 weeks, including one fatty acid dicarboxylate and one hydroxy acyl carnitine. Four urine lipid metabolites, all from the corticosteroid pathway, significantly increased at 12 weeks compared to control. **Figure 3B** shows three corticosteroids that were significantly modulated within and between groups during at least one time point.

Six stool lipid metabolites, all involved in fatty acid metabolism, increased in the rice bran + navy bean group at 12 weeks compared to control. Conversely, 14 stool lipid metabolites decreased at this time point, including two phospholipids, two phosphatidylcholines, seven lysophospholipids, and three galactosyl glycerolipids. **Figure 3C** illustrates the within and between group increase in stool octadecenedioylcarnitine and 2-hydroxysebacate at 12 weeks as well as the between group difference in pimelate at 12 weeks.

### 3.4 Stool BAs and SCFAs

After consuming rice bran + navy beans for 12 weeks, fecal excretion of the primary BA, chenodeoxycholic acid, and secondary BA, 3-oxicholic acid, was significantly increased in the intervention compared to the control group (**Table 4**). Most SCFAs trended toward increases at 12 weeks within both intervention and control groups, though no changes reached statistical significance. However, two SCFAs, butyric acid and propionic acid, were significantly higher in the control compared to the intervention group at baseline (**Table 4**). **Supplementary Table 5** shows all concentrations of BAs and SCFAs from control and intervention groups at weeks 0 and 12.

**Table 4:** Differences in stool bile acids (BA) and short chain fatty acids (SCFA) following consumption of rice bran + navy bean intervention or placebo-control for 12 weeks.

Stool metabolites	Control (n = 8) (Fibersol^®^-2)		Intervention (n = 9) (Rice bran+Navy beans)		Control Intervention	
	Week 0	Week 12	Week 0	Week 12	Week 0	Week 12
Primary BAs	mean + standard deviation		mean + standard deviation		p-value	p-value
Cholic Acid	30.1 ± 33.7	27.5 ± 15.1	81.6 ± 129.9	137.4 ± 242.8	0.695	0.165
Taurocholic Acid	0.8 ± 0.6	1.3 ± 1.5	1.3 ± 1.1	1.3 ± 1.5	0.316	0.673
Glycocholic Acid	4.3 ± 8.4	1.2 ± 0.8	2.9 ± 3	3.5 ± 5.1	0.704	0.380
Chenodeoxycholic Acid	16.5 ± 15.8	5.4 ± 6.5	42.8 ± 73.8	50.7 ± 104.1	0.909	**0.005**
Glycochenodeoxycholic Acid	5.1 ± 4.3	3.4 ± 2.5	5.5 ± 6.0	6.5 ± 8.9	0.635	0.683
Secondary BAs						
Deoxycholic Acid	481.1± 245.1	470.2 ± 322.3	404.2 ± 364.2	328.1 ± 364.2	0.257	0.314
Ursodeoxycholic Acid	23.5 ± 22.9	19.2 ± 14.0	25.0 ± 16.9	23.3 ± 13.0	0.751	0.512
Lithocholic Acid	1433.4 ± 734.2	2110.5 ± 2698.9	1442.7 ± 1345.8	1644.7 ± 2590.5	0.395	0.243
Nutriacholic Acid	214.7 ± 260.7	213.6 ± 155.8	162.1 ± 458.3	148.0 ± 96.4	0.447	0.671
7alpha-Hydroxy-3-oxo-5beta-cholanoic Acid	30.7 ± 31.4	40.3 ± 26.4	3138.6 ± 11184.6	30.2 ± 20.6	0.585	0.454
Hyodeoxycholic Acid	20.6 ±15.9	23.4 ± 15.3	45.2 ± 44.2	38.9 ± 40.1	0.216	0.412
3-Oxocholic Acid	1.6 ± 1.5	1.1 ± 0.9	4.3 ± 3.9	17.3 ± 14.6	0.319	**0.022**
3alpha-6beta-7beta-trihydroxycholenoic acid	16.7 ± 12.1	21.2 ± 8.9	16.5 ± 11.5	24.7 ± 9.3	0.713	0.939
Glycodeoxycholic Acid	5.1 ± 4.3	3.4 ± 2.5	5.5 ± 6.0	6.5 ± 8.9	0.969	0.580
Taurodeoxycholic Acid	0.9 ± 0.6	1.5 ± 1.5	5.0 ± 13.1	0.9 ± 0.7	0.363	0.679
Ursodeoxycholic Acid	23.5 ± 22.9	19.2 ± 13.9	25.0 ± 16.9	23.3 ± 13.1	0.751	0.512
3beta-hydroxy-5- cholenoic Acid	2.9 ± 5.3	0.7 ± 0.4	1.81 ± 2.7	4.79 ± 9.7	0.487	0.482
Sulfolithocholic Acid	28.1 ± 25.3	40.9 ± 84.9	64.5 ± 118.9	26.5 ± 29.1	0.619	0.496
SCFAs						
Butyric Acid	588.4 ± 455.6	1375.8 ± 2275.1	326.9 ± 206.4	467.7 ± 312.1	**0.018**	0.271
Propionic Acid	872.2 ± 555.9	1053.8 ± 529.4	406.4 ± 139.4	518.8 ± 438.8	**0.005**	0.211
Isobutyric Acid	97.9 ± 94.1	167.3 ± 243.9	83.9 ± 93.8	82.0 ± 93.4	0.352	0.478
Isovaleric Acid	92.3 ± 94.4	154.4 ± 217.7	79.8 ± 104.2	75.9 ± 87.6	0.367	0.439
Valeric Acid	114.4 ± 90.2	296.3 ± 540.3	78.9 ± 94.7	85.4 ± 93.6	0.058	0.212
Acetic Acid	2693.1 ± 1453.4	3387.2 ± 1509.0	2027.7 ± 1658.6	2038.6 ± 2039.5	0.188	0.542

BA, bile acid; SCFA, short chain fatty acids.

Values are presented as ug/ul (mean + standard deviation); statistically significant p-values are bolded (p<0.05).

### 3.5 Stool microbiome community structure

**Figure 4:**
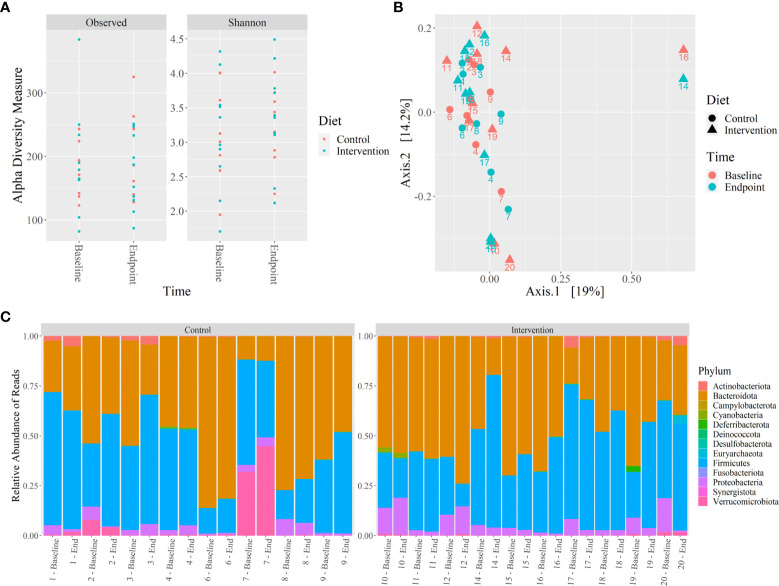
Microbiome 16S rRNA analysis of control and intervention participants at baseline and 12 weeks. **(A)** Alpha diversity analysis by Shannon and Observed diversity metrics, **(B)** Beta diversity analysis by PCoA using coordinates from unweighted Unifrac distances, **(C)** Relative abundance of microbiota phylum using 16S rRNA sequencing reads from stool. No significant differences were identified (*p ≤ 0.05*). .

No significant differences were observed in the microbiome alpha and beta diversity between control and intervention participants at baseline or at 12 weeks. The alpha diversity clustering analyzed by Shannon and Observed diversity metrics is shown in **Figure 4A**. Principal component analysis (PCoA) using the unweighted Unifrac distance showed no clustering of the beta diversity by study group or timepoint, as shown in **Figure 4B**. Phylum level relative abundance comparison did show small differences in original taxonomic units (OTU) between baseline and 12 weeks for control and intervention participants, shown in **Figure 4C**. Phyla *Firmicutes*, *Actinobacteriota*, *Proteobacteriota*, *Verrumicrobiota*, and *Bacterioidota* all showed over 1% of change from baseline to 12 weeks for at least one participant. The percent change in relative abundance for all participants for these phyla are shown in **Supplementary Table 6**.

## 4 Discussion

In this randomized, single-blinded, dietary intervention pilot study of 20 adults at risk for CRC, increased rice bran + navy bean consumption for 12 weeks showed modulation of host and gut microbial metabolism, as demonstrated by changes in plasma, urine, and stool metabolite profiles. This is the first study to evaluate the combined effects of these two functional foods on amino acid and lipid metabolites across several biological matrices and the effect on microbiome community structure and function in stool. The observed changes align with previous evaluation of metabolite profiles after controlled dietary intervention with either rice bran or navy beans (41, 42). The emergence of new metabolite changes following the combined rice bran + navy beans intake may support potential synergy when compared to the placebo fiber supplement as a control. Similarities across studies provide confidence in results and offer insights into potential biomarkers of dietary intake as well as mechanisms of action. Importantly, many of the identified metabolites have been previously associated with anticarcinogenic mechanisms, suggesting combined intake of rice bran + navy beans may reduce future risk of CRC.

When comparing the present results from the combined rice bran + navy bean intervention to our prior intervention with rice bran alone, several similarities in amino acid metabolite profiles were identified. In our previous analysis of a four-week randomized, controlled intervention of dietary rice bran, within and between group differences were noted in several plasma and urine amino acids (41). Urine N-methylleucine showed a 3.37-fold difference between intervention and control after consumption of rice bran alone. Similar between-group differences were noted in urine N-methylleucine at 6 weeks (5.64-fold difference) and 12 weeks (24.29-fold difference) as well as in stool at 12 weeks (3.62-fold difference) in the present combined rice bran + navy bean intervention. Phenol sulfate was the only amino acid metabolite observed within the rice bran intervention group that was significantly increased in both plasma and urine at 2 weeks and 4 weeks. In the present study, phenol sulfate showed between-group differences in plasma at 6 weeks (2.40-fold difference) and in urine at 6 weeks (2.50-fold difference) and 12 weeks (1.46-fold difference). Though consistency between studies was noted, the role of N-methylleucine and phenol sulfate in modulating CRC risk remains unknown, indicating a need for further study.

Comparison between the current study and our prior intervention with navy beans alone also yielded similar results for amino acid metabolites of interest. Our team has additionally demonstrated within and between group increases in several plasma and urine amino acids after four weeks of a randomized, controlled intervention of dietary navy beans (42). In plasma, we previously observed increases in S-methylcysteine, 2,3-dihydroxy-2-methylbutyrate, and pipecolate, and in urine, increases in N2,N5-diacetylornithine. These metabolites were similarly increased after combined rice bran + navy bean intervention in the present study. Indeed, in the current analysis, S-methylcysteine and pipecolate were increased within the intervention group and between intervention and control in both plasma and urine at 12 weeks. Similar to intervention with navy bean only, N2,N5-diacetylornithine was increased in plasma and urine within the intervention group at both 6 and 12 weeks. Results from this study also showed between-group differences in this metabolite in plasma and urine at both time points. Increases in 2,3-dihydroxy-2-methylbutyrate were similarly noted between intervention and control groups in both plasma and urine at 12 weeks in this study as well as in response to four weeks of navy bean consumption. Several groups have now identified these compounds as part of the dry bean metabolome, suggesting increases in navy bean intake are responsible for increases observed across multiple biological matrices and therefore may be potential candidate biomarkers of dietary exposure (42, 57–59).

Alterations in the lipid metabolite profile were slightly different between the current study and previous analyses of plasma, urine, and stool after controlled feeding of rice bran or navy beans (24, 25). However, several novel metabolites were significantly different between groups at 6 and 12 weeks, including increased plasma lipids octadecenedioate (C18:1-DC) and 9,10-DiHOME. Several plasma lipids within the plasmalogen class were also increased in intervention compared to control at 6 weeks, though many of these alterations were not maintained at 12 weeks. Stool metabolites in the dicarboxylate pathway of fatty acid metabolism were consistently increased while metabolites involved in phospholipid metabolism as well as several phosphatidylcholines and lysophospholipids were decreased in intervention compared to control at 12 weeks.

Important to cancer control are identified amino acid metabolites, including S-methylcysteine, pipecolate, 4-methoxyphenol sulfate, and 2,3-dihydroxy-2-methylbutyrate that have demonstrated antitumor properties, highlighting the relevance to future CRC prevention efforts (58). S-methylcysteine is a sulfur-containing amino acid that has been found in cruciferous vegetables, allium vegetables, and beans. This compound has been associated with reduced risk for several types of cancer, including colon cancer (60, 61). Regarding mechanism of action, S-methylcysteine has demonstrated inhibition of enzymes, such as glutathione S-transferase and ornithine decarboxylase, that have been reported to be up-regulated in a variety of cancers (62). We have also shown increases in plasma S-methylcysteine and concomitant protection from azoxymethane (AOM) and dextran sodium sulfate (DSS)-induced tumorigenesis after human fecal transplantation of rice bran-modified human microbiota communities into mice (63). Importantly, this impact of rice-bran modified gut microbiota on CRC was independent of other foods consumed in the diet. Similarly, pipecolate, a precursor to gut microbial metabolism, has been shown to have antitumor, anti-inflammatory, and antibiotic properties in several preclinical studies (64–67). A novel finding to this study that was not observed in previous interventions of rice bran or navy beans alone was the change in plasma and urine 4-methoxyphenol sulfate levels. Previous work indicates 4-methoxyphenol sulfate suppresses tumor growth in preclinical models of AOM-induced colon carcinogenesis (68). 2,3-dihydroxy-2-methylbutyrate is an intermediate in branched chain amino acid metabolism and a known product of gut microbial metabolism that may have anti-inflammatory properties (42). In previous work, we have shown greater BMI is associated with greater number of polyps and lower number of amino acids detected in colon tissue (69). Though the complete mechanism of action is unknown, decreased levels have also been associated with obesity in preclinical models and are responsive to prebiotic supplementation, suggesting potential metabolic benefit and meriting additional follow-up (70).

Lipid metabolites modulated by rice bran + navy bean consumption that have demonstrated potential anticarcinogenic properties in previous studies include octadecenedioate and 9,10-DiHOME, which are both involved in fatty acid metabolism, as well as several compounds involved in glucocorticoid metabolism. Octadecenedioate has been investigated for its relationship to CRC in fecal metabolomics analyses, demonstrating a higher prevalence in controls compared to CRC cases, though additional follow-up is warranted (71). The fatty acid 9,10-DiHOME is known to recruit neutrophils to inflamed sites during an innate immune response to tissue damage and/or infection (72). Additionally, after 12 weeks of consuming rice bran + navy beans, several metabolites in the corticosteroid pathway were significantly increased compared to control, including urine cortisol, cortisone 21-sulfate, and tetrahydrocortisol sulfate. Clinical studies have shown several medicinal plants used to treat inflammation contain compounds which chemically resemble steroids in structure, supporting their potential role as anti-inflammatory agents (73). For example, *Glycyrrihiza glabra* is a plant containing chemical constituents having steroidal structure that is reported to act similar to cortisone to reduce inflammation (74, 75). Further, glucocorticosteroids have been used widely in conjunction with other treatments for patients with cancer (76, 77). Evidence demonstrates that glucocorticoids act to inhibit solid tumor growth *via* downregulation of tumor-associated inflammation/angiogenesis (78). More studies are warranted to determine if plant steroids (phytosterols) from rice bran and navy beans serve as effective anti-inflammatory agents.

In this study, we also observed increased carnitines in stool and plasma after 12 weeks in intervention compared to placebo-control. Carnitine metabolism has been evaluated for its anticancer effects, and previous studies indicate direct or indirect activity with dietary fibers to decrease iron absorption and protect against oxidative stress, potentially through gut microbial composition and function (79, 80). Over 75% of the total body carnitine originates from food sources and is correlated with plasma carnitine concentrations (81). To our knowledge, this is the first study to report diet-derived octadecenedioylcarnitine increasing in plasma and stool after consumption of rice bran + navy beans, warranting follow up for association with reducing CRC risk.

Our previous analyses suggest that although activity levels remained unchanged after physical activity education and the combined rice bran + navy bean dietary intervention, stool BAs and SCFAs were cross-sectionally associated with physical activity within this cohort independent of fiber intake (47, 49). To our knowledge, this is the first analysis of changes in BAs and SCFAs after a combined rice bran + navy bean intervention. Significant increases in the stool secondary BA, 3-oxocholic acid, and primary BA, chenodeoxycholic acid, was observed among a subset of participants in the intervention group compared to control. Studies have shown 3-oxocholic acid is associated with probiotic bacterial species and enriched in non-cachectic cancer patients compared to those with cachexia, indicating a pathway through which the gut microbiome may serve as a potential target for treatment (82). However, preclinical studies have indicated that this gut-derived BA may play a role in modulating individualized response during CRC treatment, highlighting the complex relationship between the gut microbiome and CRC risk and indicating the need for additional follow up (83). No significant differences were noted between intervention and control for stool SCFAs nor microbial community structures (richness, evenness, diversity), as assessed by 16S amplicon sequencing. These results are similar to our previous interventions with rice bran or navy beans alone, which showed within-intervention changes after intake of rice bran but no differences between groups (25, 40). It is possible the dose or length of the dietary intervention was insufficient to modulate the gut microbiome, as others have demonstrated significant differences between intervention and control after a longer period of dietary intervention (84). In our trial, and as noted in several previous studies, measures of community structure were more similar within an individual than between individuals across time points and regardless of dietary intervention (85). This pilot study provided novel microbial and metabolic insights for developing rice bran and navy beans with a precision nutrition approach for reducing CRC risk.

While this study had many strengths, including a randomized, controlled design with repeated biological sampling and use of previously validated methods for metabolite and stool microbiome analyses, it is not without limitations. The small cohort size limits statistical power and generalizability of results, yet it was sufficient for a pilot trial design and supports feasibility for larger cohort investigations. Additionally, longer duration of dietary intervention with rice bran + navy beans may be needed to observe changes in microbiome community structure and metabolism. Further, incorporating randomization by BMI, pre-existing conditions, number of polyps removed, location of polyps, and/or other factors such as baseline fiber intake would strengthen conclusions. Future studies should also consider a longitudinal design with greater duration of follow up to determine impact on disease outcomes such as diagnosis of CRC, disease-free survival, and/or CRC-related mortality. In addition, other aspects of health, including quality of life, should be assessed to better define impacts of the intervention across multiple domains. To expand upon these results and address these limitations, we plan to prospectively test the effect of the combined rice bran + navy bean intervention on polyp prevention within a larger trial in individuals with Lynch syndrome.

This study is the first to test the impact of a combined rice bran + navy bean intervention on plasma, urine, and stool metabolite profiles to provide insight into potential effects on future CRC risk. Importantly, observed changes following the intervention align with previous research and may contribute to prevention of CRC recurrence through modulation of immunological and anti-inflammatory pathways. Reduction in CRC risk through alterations in dietary intakes of discrete foods provides a non-invasive and translational method to enhance and tailor dietary cancer prevention and control efforts in community settings. Further, rice bran and navy beans are affordable and accessible, thus making them ideal targets for dietary intervention in a variety of populations.

## Data availability statement

The datasets presented in this study can be found in online repositories. The names of the repository/repositories and accession number(s) can be found in the article/**Supplementary Material**.

## Ethics statement

The study protocol and written informed consent was approved by Colorado State University Research Integrity and Compliance Review Board and the University Colorado Health Cancer Center-North Institutional Review Board (Protocol #17-7464H) in accordance with the 1964 Helsinki Declaration and its 2013 amendments. The study was completed between September 2017 and December 2018. The patients/participants provided their written informed consent to participate in this study.

## Author contributions

EH: Writing-review and editing. BB: Data curation, writing-original draft, study coordinator. BP: Writing-review and editing. CS: Writing-review and editing. MB: Data curation, writing-review, and editing. HS: writing-review and editing. SS: Writing-review and editing. MT: writing-review and editing. HI: writing-review and editing. SR: Statistics. HL: Conceptualization, methodology, and project administration. ER: Conceptualization, data curation, visualization, methodology, project administration, writing-original draft, review, and editing. All authors contributed to the article and approved the submitted version.
