# Supplementary material for: Plasma, urine, and stool metabolites in response to dietary rice bran and navy bean supplementation in adults at high-risk for colorectal cancer
Source: Front Gastroenterol (Lausanne). Author manuscript; Available in PMC 2024 Mar 11. (PMC10927265; doi:10.3389/fgstr.2023.1087056)
Supplement: Supplementary Tables 1-6 [file NIHMS1967146-supplement-Supplementary_Tables_1-6.zip › Table_1_Plasma, urine, and stool metabolites in response to dietary rice bran and navy bean supplementation in adults at high-risk for colorectal canc.docx]

**Supplementary Table 1.** List of metabolites targeted for quantification in stool at baseline (week 0) and endpoint (week 12)

| **SRM Methods for LCMS Bile Acid Quantitation** | | | | | | |
| --- | --- | --- | --- | --- | --- | --- |
| **Metabolite** | **Retention Time (min)** | **Cone Voltage** | **Collision Energy** | **Precursor m/z** | **Product m/z** | **Ionization Mode** |
| Cholic acid^a^ | 1.59 | 50 | 4 | 407.3 | 407.3, 389.3 | Negative |
| Taurocholic acid^a^ | 1.06 | 50 | 50 | 514.3 | 80 | Negative |
| Taurocholic acid_d5^b^ | 1.05 | 50 | 50 | 519.3 | 124 | Negative |
| Glycocholic acid^a^ | 1.32 | 50 | 40 | 464.3 | 74 | Negative |
| Chenodeoxycholic acid^a^ | 1.94 | 50 | 30 | 391.3 | 373.3, 391.3 | Negative |
| Glycochenodeoxycholic acid and Glycodeoxycholic acid^a^ | 1.67/1.62 | 50 | 40 | 448.3 | 74 | Negative |
| Glycodeoxycholic acid-d4^b^ | 1.67 | 50 | 40 | 452.3 | 74 | Negative |
| Deoxycholic acid^a^ | 1.99 | 50 | 30 | 391.3 | 345.3, 391.3 | Negative |
| Deoxycholic acid-d4^b^ | 1.99 | 50 | 30 | 395.3 | 395.3, 349.3 | Negative |
| Lithocholic acid^a^ | 2.4 | 50 | 4 | 375.3 | 375.3, 357.3 | Negative |
| Nutriacholic acid^a^ | 1.76 | 50 | 4 | 389.3 | 389.3, 371.3 | Negative |
| 7alpha-Hydroxy-3-oxo-5beta cholanoic acid^a^ | 2.0 | 50 | 4 | 389.3 | 389.3, 371.3 | Negative |
| Hyodeoxycholic acid and Ursodeoxychoic acid^a^ | 1.64/1.62 | 50 | 4 | 391.3 | 391.3, 373.3 | Negative |
| 3-oxocholic acid^a^ | 1.58 | 50 | 4 | 405.3 | 405.3, 369.3 | Negative |
| 3alpha,6beta,7beta-Trihydroxy-5b-cholanoic acid^a^ | 1.41 | 50 | 4 | 407.3 | 407.3, 371.3 | Negative |
| Taurodeoxycholic acid^a^ | 1.44 | 50 | 50 | 498.3 | 124 | Negative |
| 3beta-hydroxy-5-cholenoic acid^a^ | 2.13 | 50 | 4 | 373.3 | 373.3, 355.3 | Negative |
| Sulfolithocholic acid^a^ | 1.87 | 50 | 40 | 455.4 | 97 | Negative |
| SIM Methods for GCMS Short Chain Fatty Acid Quantitation | | |  |  |  |  |
| Metabolite | **Retention Time (min)** | **Quantification ion m/z** |  |  |  |  |
| Butyric acid^b,c^ | 5.861 | 60 |  |  |  |  |
| Propionic acid^b^ | 4.999 | 74 |  |  |  |  |
| Isobutyric acid^b^ | 5.23 | 88 |  |  |  |  |
| Isovaleric acid^b^ | 6.29 | 60 |  |  |  |  |
| Valeric acid^b^ | 6.98 | 60 |  |  |  |  |
| Acetic acid^b^ | 4.199 | 60 |  |  |  |  |

^a^Non-labeled standards for analysis of 5 primary bile acids and 12 secondary bile acids were purchased from MetaSci, Richmond Hill, ON, Canada.

^b^Non-labeled standards for analysis of 6 short chain fatty acids and labeled standards for ^13^C_2_-acetic acid . Labeled standards for taurocholic acid-d5, and deoxycholic acid-d4 were purchased from Sigma-Aldrich, St. Louis, MO.

^c^Labeled standards for ^13^C_4_-sodium butyrate were purchased from Santa Cruz Biotechnology, Dallas, TX.
