# Supplementary material for: Plasma, urine, and stool metabolites in response to dietary rice bran and navy bean supplementation in adults at high-risk for colorectal cancer
Source: Front Gastroenterol (Lausanne). Author manuscript; Available in PMC 2024 Mar 11. (PMC10927265; doi:10.3389/fgstr.2023.1087056)
Supplement: Supplementary Tables 1-6 [file NIHMS1967146-supplement-Supplementary_Tables_1-6.zip › Table_6_Plasma, urine, and stool metabolites in response to dietary rice bran and navy bean supplementation in adults at high-risk for colorectal canc.docx]

**
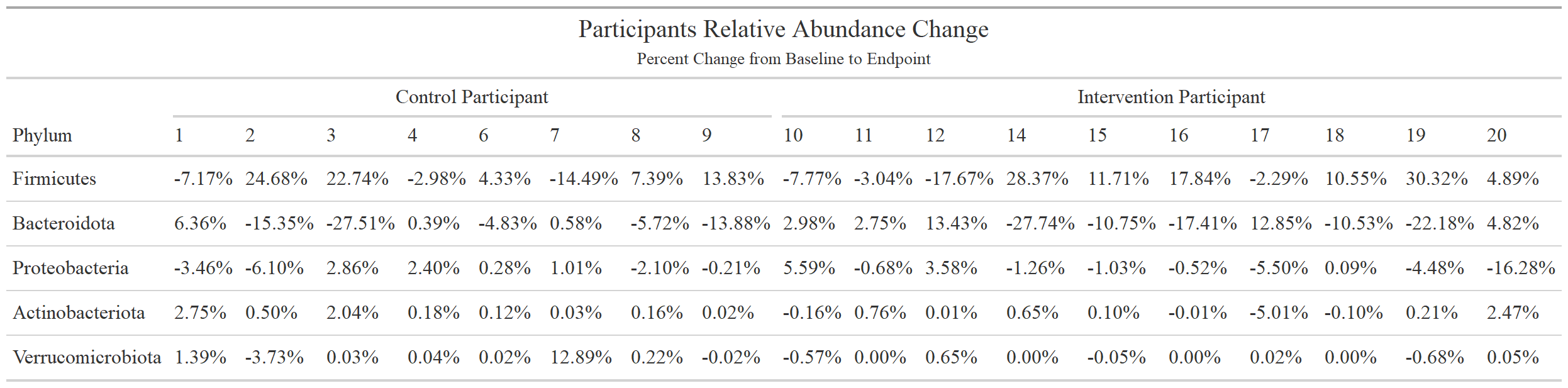
Supplementary Table 6**. Percentage of change from baseline to 12 weeks in relative abundance of OTUs at the Phylum level for control and intervention groups. (n =18)
